# Periarticular cocktail injection is more useful than nerve blocks for pain management after anterior cruciate ligament reconstruction

**DOI:** 10.1016/j.asmart.2024.03.001

**Published:** 2024-04-03

**Authors:** Tomoyuki Kanayama, Junsuke Nakase, Rikuto Yoshimizu, Yoshihiro Ishida, Yusuke Yanatori, Yu Arima, Naoki Takemoto

**Affiliations:** a Department of Orthopedic Surgery, Graduate School of Medical Sciences, Kanazawa University, Kanazawa, Japan

**Keywords:** Anesthesia, Anterior cruciate ligament reconstruction, Autografts, Femoral neuropathy, Nerve block pain management

## Abstract

**Background:**

Anterior cruciate ligament (ACL) reconstruction is commonly associated with moderate-to-severe postoperative pain. Notably, various pain control strategies, a femoral nerve block (FNB) with a lateral femoral cutaneous nerve block (LFCNB), adductor canal block (ACB) with LFCNB, or periarticular cocktail injection (PI), have been investigated. However, no studies compare the effects of FNB with LFCNB, ACB with LFCNB, and PI for pain control after ACL reconstruction. This study aimed to evaluate the impact of FNB with LFCNB, ACB with LFCNB, and PI for pain relief in the early postoperative period after ACL reconstruction.

**Methods:**

This retrospective controlled clinical trial enrolled 299 patients who underwent primary ACL reconstruction at our hospital between April 2016 and October 2022. We categorized these cases into groups based on the use of PI (PI group), FNB with LFCNB (FNB group), and ACB with LFCNB (ACB group) for pain management. We selected 40 cases each, with matched age, sex, and body mass index (BMI) from each group, resulting in 120 cases for analysis. In the FNB and ACB groups, 0.75% ropivacaine 15 ml was injected under ultrasound guidance preoperatively. In the PI group, a mixture of 0.75% ropivacaine 20 ml, normal saline 20 ml, and dexamethasone 6.6 mg was injected half at the start of surgery and the rest just before wound closure. Patient demographics (age, sex, height, body weight, and BMI) and surgical data (the requirement for meniscal repair, operative time, and tourniquet inflation time) were analyzed. After ACL reconstruction, patients' numerical rating scale pain scores (NRS) (0-10) were recorded at 30 min and 4, 8, 12, 24, 48, and 72 h postoperatively. NRS were then compared among the three groups using analysis of variance. In addition, within each group, these data were compared between the NRS ≥7 and NRS ≤6 groups using a *t*-test.

**Results:**

There were no significant differences in patient demographics and surgical data. Pain scores were significantly higher in the PI group than in the FCB and ACB groups 30 min postoperatively, but they were lower at 12, 24, 48, and 72 h postoperatively. In the FNB group, there were no significant differences in the demographic and surgical data by NRS pain score. In the ACB group, the number of men was significantly higher in the NRS ≥7 group than in the NRS ≤6 group (p = 0.015). In the PI group, tourniquet inflation time was significantly longer in the NRS ≥7 group than in the NRS ≤6 group (p = 0.008).

**Conclusions:**

Following ACL reconstruction using a hamstring autograft, periarticular cocktail significantly reduced early postoperative pain compared with nerve block combinations.

## 1 Introduction

An anterior cruciate ligament (ACL) tear is a common injury in sports medicine, for which reconstruction is a commonly used treatment option. Notably, ACL reconstruction is usually associated with moderate-to-severe postoperative pain.^1^^,^^2^ Postoperative pain control after ACL reconstruction has received considerable attention, as it is vital to improving patient satisfaction and outcomes^3^ and reducing healthcare costs.^4^ Notably, several pain control strategies, such as administering intra-articular injection,^5^ periarticular injection (PI),^6^^,^^7^ and peripheral nerve blocks, especially the femoral nerve and adductor canal blocks,^8^^,^^9^ have been investigated. In randomized clinical trials, the femoral nerve block (FNB) and adductor canal block (ACB) provided similar and effective analgesia following ACL reconstruction.^9^^,^^10^ However, these two blocks control pain mainly in the leg's anterior and medial aspects.^11^ The lateral femoral cutaneous nerve (LFCN) supplies sensation to the lateral portion of the thigh, and the corresponding nerve block can address pain in this area in patients who have undergone ACL reconstruction.^1^^,^^5^^,^^12^ Oshima et al.^13^ reported that combining an ACB with an LFCN block (LFCNB) significantly reduced postoperative pain in the early phase compared with pain reduction after administering a FNB combined with an LFCNB.

A periarticular injection is a typical, rapid, and safe treatment for postoperative pain control after knee replacement surgery. In ACL reconstruction, significantly superior pain scores are observed with periarticular injection than with an FNB, with no increase in complication rates.^6^ However, no reports have compared the effects of the FNB combined with an LFCNB, ACB combined with an LFCNB, and PI for pain control after ACL reconstructive surgery. Therefore, this study aimed to investigate the effects of these injections on pain in the early postoperative period after ACL reconstruction. We hypothesized that PI would provide better pain relief than the combinations of FNB with LFCNB or ACB with LFCNB.

## 2 Materials and methods

### 2.1 Study design and participants

This retrospective controlled clinical trial was approved by the ethics committee of our hospital (IRB No. 1842-3), and the study follows the principles of the Declaration of Helsinki. All patients included in the study provided written informed consent before participation.

This study included 299 patients who underwent primary ACL reconstruction at our hospital between April 2016 and October 2022. Patients received a FNB combined with a LFCNB between April 2016 and December 2019 (FNB group), an ACB combined with a LFCNB between January 2020 and September 2021 (ACB group), and a periarticular multi-drug cocktail injection between October 2021 and October 2022 (PI group). Each group comprised patients matched for age, sex, and body mass index (BMI). We excluded patients who had undergone revision surgery for ACL re-tears and concurrent meniscal tears requiring inside-out repair or had alcohol or drug abuse history, concurrent ligament injuries requiring repair, and cartilage injury requiring microfracture or grafting.

The numerical rating scale (NRS) pain score is based on how much the pain hinders patients' general functioning. Notably, ≤3, 4–6, and ≥7 scores correspond to mild, moderate, and severe pain, respectively.^14^ To further investigate the efficacy of the pain control method used in reducing severe pain, the NRS pain scores at 30 min postoperatively were used to classify the patients into the NRS score ≤6 (NRS ≤6) or ≥ 7 (NRS ≥7) groups. A NRS pain score ≤6 indicated moderate pain, whereas ≥7 indicated severe pain. ACL reconstruction was performed using a hamstring tendon autograft and the anatomic single-bundle technique.^15^ One surgeon performed the procedures using a tourniquet at a single center. Patients regularly received 200 mg of celecoxib orally in the morning and evening for 1 week postoperatively. Pain was routinely assessed, and analgesics were administered as required at the physician's discretion. The patients were discharged 1 week postoperatively with crutches and were recommended only partial weight bearing.

### 2.2 Interventions

In the FNB and ACB groups, an experienced orthopedist administered the nerve blocks following general anesthesia 30 min preoperatively. A high-frequency linear-array ultrasound transducer was used. For the FNB, the ultrasound transducer was placed at the level of the inguinal crease to help visualize the fascia iliaca, femoral artery, and femoral nerve. A 60-mm 25-gauge needle was inserted lateromedially towards the femoral nerve and deep into the fascia iliaca using an in-plane technique.^12^

For the ACB, the transducer was placed in the transverse direction on the medial side of the middle thigh to identify the femoral artery anteromedially, with the sartorius muscle in the short-axis view. A 60-mm 25-gauge needle, using an out-of-plane technique, was inserted anterolateral to the femoral artery and just deep into the posterior fascia of the sartorius muscle.^16^ For both the FNB and ACB, 10 ml of 0.75% ropivacaine was injected.

The transducer was placed parallel to the inguinal ligament and distal to the anterior superior iliac spine for the LFCNB. The LFCN is visualized as a hypoechoic fat-filled flat tunnel formed by a layer of fascia latae between the sartorius muscle medially and the tensor fascia latae muscle laterally. A 38-mm 25-gauge needle was inserted laterally and medially to position the needle tip around the LFCN using an in-plane approach. Subsequently, 5 mL of 0.75% ropivacaine was injected.^17^^,^^18^

For the PI group, the operative orthopedic surgeon administered local infiltration analgesia with 20 mL of 7.5 mg/mL, ropivacaine, 20 ml of saline, and 6.6 mg of 6.6 mg/2 mL dexamethasone (total, 42 mL). Patients received half the periarticular injection at the start of the surgical procedure and half before wound closure. Before commencing surgery, 11 mL of this solution was injected, using a 32-mm 23-gauge needle, into the infrapatellar fat pad: 5 mL was injected into the portal's subcutaneous tissue and 5 mL into the incision's subcutaneous tissue at the hamstring harvest site. Before skin closure, 5 mL of the solution was injected into every visible region around the hamstring harvest site, 11 mL was injected around the hamstring muscle-tendon transition area, and 5 mL was injected into the medial and lateral synovial capsule above the meniscus using a 60-mm 22-gauge needle. No bolus periarticular or intra-articular injections were administered beyond the day of surgery.

### 2.3 Data collection

Patient demographics (age, sex, height, body weight, and BMI) were evaluated. The requirement for meniscal repair, operative time, and tourniquet inflation time was also assessed as a part of surgical data. For clinical evaluation following ACL reconstruction, the pain was recorded on a NRS^3^ from 0 to 10 at 30 min and 4, 8, 12, 24, 48, and 72 h after returning to the hospital room. Numerical rating scores for knee pain were recorded by nurses who verbally questioned the patients regarding their pain. The administration and use of analgesic suppositories (50 mg diclofenac sodium or 300 mg acetaminophen) were evaluated for each patient. Per the patient's wishes, suppositories were used at ≥4 h intervals. Acetaminophen was administered if the patient was younger than 13 or weighed <45 kg.

### 2.4 Statistical analyses

The sample size was determined by power analysis based on data from a previously reported pilot study using G*Power (ver. 3.1.9.3, Heinrich-Heine-Universität Düsseldorf, Düsseldorf, Germany) with the NRS pain score as the primary variable. Referring to previous studies,^8^^,^^13^ we set the accepted alpha error as 0.05, the beta error as 0.2 (power: 80%), the mean difference between the three groups as 1, and the standard deviation as 1.5. The required sample size for this study was 111 patients (37 in each group). All statistical analyses were performed using SPSS version 29.0 (IBM Corp. Armonk, NY, USA). Differences in patient demographics, surgical data, and clinical evaluations among the three groups were assessed using analysis of variance. In addition, within each group, these data were compared between the NRS ≥7 and NRS ≤6 groups using Student's t-test. Statistical significance was set at p < 0.05.

## 3 Results

**Fig. 1:**
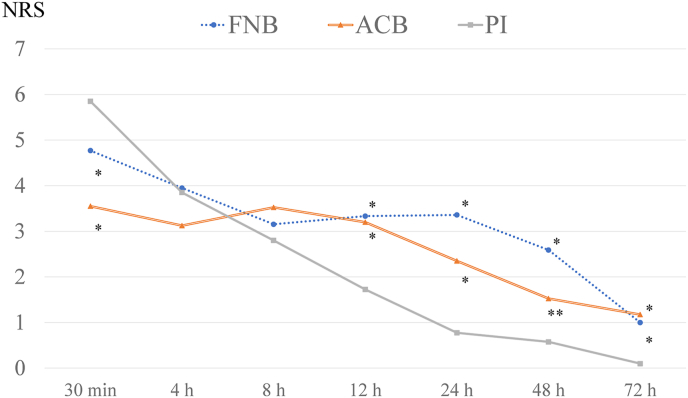
Postoperative NRS changes in the three groups Pain scores were compared between the FNB, ACB, and PI groups. *: *P* < 0.01, **: *P* < 0.05 Abbreviations: NRS, numerical rating scale; FNB, femoral nerve block; ACB, adductor canal block; PI, periarticular injection.

This study included 47 men and 73 women; with a mean patient age of 25.3 ± 13.0 years, and a mean BMI of 23.1 ± 3.8 kg m^−2^ at the time of surgery. The patient demographics and surgical data are presented in Table 1. The final study included 120 participants split equally between the three groups (each group comprised 40 patients matched for age, sex, and BMI). There were no significant differences in age, sex, height, body weight, and BMI among the three groups. In addition, there were no significant differences in the requirement for meniscal repair, operative time, tourniquet inflation time, and the number of suppositories used among the three groups. Pain scores were significantly higher in the PI group than in the FNB and ACB groups at 30 min (FNB group, p = 0.008; ACB group, p = 0.003) but significantly lower at 12 (FNB group, p < 0.001; ACB group, p = 0.001), 24 (FNB group, p < 0.001; ACB group, p < 0.001), 48 (FNB group, p < 0.001; ACB group, p = 0.033), and 72 h (FNB group, p = 0.002; ACB group, p < 0.001) (Fig. 1). No complications were observed in any group at the end of the study.

**Table 1:** Comparison of patient demographic data between the groups.

	FNB group	ACB group	PI group	*P* value
	n = 40	n = 40	n = 40	
Age (years)	22.4 ± 12.4	26.4 ± 13.7	26.2 ± 12.2	a 0.353
				b 0.405
				c 0.995
Male/female (knees)	16/24	18/22	13/27	a 0.932
				b 0.722
				c 0.494
Height (cm)	163.5 ± 7.9	165.4 ± 8.5	166.4 ± 7.2	a 0.544
				b 0.234
				c 0.828
Body weight (kg)	61.5 ± 11.6	64.0 ± 12.4	16.3 ± 13.6	a 0.658
				b 0.590
				c 0.994
BMI (kg/m^2^)	22.9 ± 3.6	23.3 ± 3.6	23.2 ± 4.2	a 0.898
				b 0.968
				c 0.979
Presence of meniscal repair (knees)	21	22	23	a 0.942
				b 0.847
				c 0.973
Operation time (min.)	105.6 ± 22.1	102.8 ± 21.4	106.0 ± 25.2	a 0.853
				b 0.997
				c 0.809
Tourniquet inflation time (min.)	90.6 ± 19.5	89.9 ± 19.3	85.7 ± 18.7	a 0.985
				b 0.484
				c 0.587
Number of suppository use (times)	1.0 ± 0.8	1.0 ± 0.9	0.9 ± 1.2	a 1.000
				b 0.943
				c 0.942

Data are presented as mean ± standard deviation.

Abbreviations: FNB, femoral nerve block; ACB, adductor canal block; PI, periarticular injection; BMI, body mass index.

a Comparison of FNB and ACB groups.

b Comparison of FNB and PI groups.

c Comparison of ACB and PI groups.

In the FNB group, there were no significant differences in the demographic and surgical data by NRS pain score (Table 2). In the ACB group, the number of men was significantly higher in the NRS ≥7 group than in the NRS ≤6 group (p = 0.015; Table 3). In the PI group, tourniquet inflation time was significantly longer in the NRS ≥7 group than in the NRS ≤6 group (p = 0.008; Table 4).

**Table 2:** Comparison between NRS ≤6 and NRS ≥7 in the FNB group.

	NRS ≤6	NRS ≥7	*P* value
	n = 26	n = 14	
Age (years)	22.2 ± 13.2	22.9 ± 11.4	0.428
Male/female (knees)	12/14	4/10	0.124
Height (cm)	164.1 ± 8.9	162.5 ± 5.7	0.275
Body weight (kg)	60.2 ± 11.6	63.8 ± 11.7	0.176
BMI (kg/m^2^)	22.2 ± 3.1	24.2 ± 4.2	0.117
Presence of meniscal repair (knees)	11	9	0.054
Operation time (min.)	105.4 ± 20.2	106.0 ± 25.8	0.468
Tourniquet inflation time (min.)	87.9 ± 17.1	95.5 ± 22.9	0.124
Number of suppository use (times)	0.84 ± 0.8	1.29 ± 0.8	0.054

Data are presented as mean ± standard deviation.

Abbreviations: NRS, numerical rating scale; FNB, femoral nerve block; BMI, body mass index.

**Table 3:** Comparison between NRS ≤6 and NRS ≥7 in the ACB group.

	NRS≤6	NRS≥7	P value
	n = 29	n = 11	
Age (years)	26.0 ± 13.9	27.5 ± 13.9	0.387
Male/female (knees)	10/19	8/3	0.015
Height (cm)	166.4 ± 8.4	162.6 ± 8.6	0.102
Body weight (kg)	65.2 ± 13.2	60.7 ± 9.4	0.153
BMI (kg/m^2^)	23.5 ± 3.7	23.0 ± 3.4	0.357
Presence of meniscal repair (knees)	18	4	0.078
Operation time (min.)	104.0 ± 18.9	99.7 ± 27.6	0.289
Tourniquet inflation time (min.)	92.5 ± 19.8	83.4 ± 17.1	0.093
Number of suppository use (times)	0.90 ± 1.0	1.3 ± 0.5	0.13

Data are presented as mean ± standard deviation.

Abbreviations: NRS, numerical rating scale; ACB, adductor canal block; BMI, body mass index.

**Table 4:** Comparison between NRS ≤6 and NRS ≥7 in the PI group.

	NRS≤6	NRS≥7	P value
	n = 22	n = 18	
Age (years)	23.8 ± 11.4	29.0 ± 12.9	0.093
Male/female (knees)	7/15	6/12	0.461
Height (cm)	166.1 ± 6.4	166.7 ± 8.4	0.382
Body weight (kg)	62.5 ± 9.8	66.5 ± 17.2	0.182
BMI (kg/m^2^)	22.7 ± 3.3	23.8 ± 5.1	0.206
Presence of meniscal repair (knees)	15	8	0.069
Operation time (min.)	101.0 ± 24.9	112.1 ± 24.8	0.085
Tourniquet inflation time (min.)	79.4 ± 17.4	93.4 ± 17.7	0.008
Number of suppository use (times)	0.9 ± 1.6	0.9 ± 0.7	0.465

Data are presented as mean ± standard deviation.

Abbreviations: NRS, numerical rating scale; PI, periarticular injection; BMI, body mass index.

## 4 Discussion

This study found that after single-bundle ACL reconstruction using a hamstring autograft, PI of a cocktail was significantly more effective for pain control in the early postoperative period than the combination of nerve blocks administered.

The joint pain management guidelines of five American societies (American Society of Hip and Knee Surgeons, American Orthopaedic Society, Hip Society, Knee Society, and American Society of Regional Anesthesia and Pain) strongly recommended using PI due to the very high standardized mean difference of −0.53 (95% CI: −0.8 to −0.25) for pain relief with periarticular injections compared with that with the placebo.^19^ PI involves a combination of multiple agents, including local anesthetics, steroids, and opioids, in various proportions. As multiple sensory nerves innervate the peripheral joints, uniform periarticular penetration by local anesthesia can compensate for areas not covered by peripheral nerve blocks. The guidelines^19^ also strongly recommend using long-acting anesthetics but suggest moderation in the case of steroids. However, morphine use is strongly discouraged as it does not provide additional analgesia and may cause vomiting and nausea. Therefore, this study used a mixture of long-acting anesthetics and steroids. This mixture provided beneficial results regarding patient wellness following treatment, as no vomiting or nausea were encountered.

When local anesthesia is administered, the pain caused by a tourniquet increases in intensity.^20^ Tourniquet pain is a nociceptive pain caused by peripheral nociceptors and direct central nervous system stimulation.^21^ Duration of contraption use is directly proportional to the frequency and intensity of tourniquet pain. Therefore, minimizing the period of tourniquet use is recommended.^22^ This study found that the greater the pain in the PI group, the longer the tourniquet inflation time (P = 0.008, effect size = 0.80, power = 0.79). However, the tourniquet inflation time or pain level did not significantly differ between the FNB and ACB groups. The local anesthesia's ineffectiveness for tourniquet pain may be due to the following: 1) its inability to cover a large area; 2) the FNB being performed proximal to the tourniquet ejection site; or 3) the ACB producing anesthetic effects by backflow of anesthesia through the sheath. Tourniquet blocks and other pain-relieving modalities are recommended when regional anesthesia is used.^3^ In the future, it may be necessary to consider reducing tourniquet inflation time and combining periarticular injections with nerve blocks.

Peripheral nerve blocks are used for pain control following ACL reconstruction. The FNB has traditionally been the ACL's peripheral nerve block of choice because it has reliably provided adequate analgesia.^23^ However, the FNB may cause block-related weakness of knee extensor strength,^24^^,^^25^ delaying the return to sports after ACL reconstruction.^26^ The ACB has attracted attention as an alternative regional block to the FNB because it reduces muscle weakness as a non-motor nerve regional block.^12^^,^^15^^,^^27^, ^28^, ^29^ In healthy volunteers, Kwofie et al.^12^ identified that ACB did not significantly affect quadriceps motor function compared with the FNB at 30 and 60 min after block administration.

Furthermore, in their randomized controlled trial, Abdallah et al.^27^ demonstrated that during ACL reconstruction, compared with FNB, ACB led to superior isometric quadriceps strength at 45 min after block administration. Bailey et al.^28^ also reported that ACB administration led to fewer deficits in quadriceps muscle activation at 24 h and 2 weeks than did FNB administration, with no significant differences in quadriceps muscle strength at 6 months after ACL reconstruction. In addition, in their systematic review, Edwards et al.^29^ suggested that compared with FNB, ACB was superior in preserving quadriceps function in the early postoperative period after ACL reconstruction. Notably, a recent meta-analysis reported that FNB was superior to local infiltration anesthesia for analgesia after ACL reconstruction regarding the pain relief achieved.^30^ However, this study did not compare FNB with ACB, and the solutions used for local infiltration anesthesia were not standardized. To ensure our study covered these factors, we evaluated multiple methods, including both FNB and ACB and the solutions used were standardized. A meta-analysis of five American societies’ joint pain management guidelines, a mixture of long-acting anesthetics and steroids, reported that local infiltration anesthesia showed better pain relief than FNB.^19^ In the meta-analysis by Kirkham et al.,^30^ which addressed muscle strength, no complications of muscle weakness were reported. The mixture of steroids with local infiltration anesthesia was thought to provide better pain relief than nerve blocks. Therefore, we included this mixture in our study to ensure elevated results.

LFCN supplies sensation to the lateral aspect of the thigh, and the corresponding nerve block can address pain in the lateral aspect of the thigh in patients undergoing ACL reconstruction.^8^^,^^11^^,^^17^ Reportedly, after ACL reconstruction, analgesia of the lateral side supplied by the LFCN significantly reduces postoperative pain compared with that of the posterior area by local anesthetic infiltration of the interspace between the popliteal artery and capsule of the posterior knee.^8^ Oshima et al. reported that after single-bundle ACL reconstruction using a hamstring autograft, the combination of an ACB with an LFCNB was significantly more effective at reducing pain in the early postoperative period compared with the combination of an FNB with an LFCNB.^13^ However, the present study found that PI was superior to nerve blocks in reducing pain.

This study had some limitations. First, the postoperative analgesic area was not evaluated. Second, the study was neither anonymized nor randomized. This study design may have influenced the current study's results, as knowledge of the pain control method used may have influenced how the surgeon asked about the degree of pain. However, nurses in this study were asked about the NRS pain scores, and this influence was considered negligible. Finally, this study did not include a control group; therefore, we could not confirm the actual therapeutic effects of the nerve blocks or local anesthetic infiltration. Despite these limitations, this study's strength lies in the fact that it was performed by the same surgeon using the same technique to administer the block and periarticular injections, reducing the variation between the patients and treatments. The present study's findings are crucial for improving pain control, patient satisfaction, and patient outcomes after ACL surgery in the clinical setting. Depending on the results of further studies in addressing the present study's limitations, physicians may take the evidence provided within this study to advocate for the use of PI over nerve block injection to relieve post-ACL reconstruction pain, affording their patients an improved quality of life. This finding could lead to improved patient postoperative care and quality of life. Further studies are required to address the effect of varying the postoperative analgesic injection site, reducing tourniquet inflation time, and combining periarticular and block injections.

## 5 Conclusion

After single-bundle ACL reconstruction with hamstring autograft, periarticular cocktail injection significantly reduced early postoperative pain compared with nerve block combinations.

## Author contributions

The study was designed by TK and JN. All data were analyzed by YY. Data interpretation and manuscript preparation were undertaken by all authors. All authors read and approved the final manuscript.

## Data availability statement

The datasets are available from the corresponding author upon reasonable request.

## Declaration of competing interest

The authors did not receive support from any organization for the submitted work.

The authors have no conflicts of interest to declare that are relevant to the content of this article.

All authors certify that they have no affiliations with or involvement in any organization or entity with any financial interest or non-financial interest in the subject matter or materials discussed in this manuscript.
