# Supplementary material for: Periarticular cocktail injection is more useful than nerve blocks for pain management after anterior cruciate ligament reconstruction
Source: Asia Pac J Sports Med Arthrosc Rehabil Technol. 2024 Apr 3;36:45–9. doi: 10.1016/j.asmart.2024.03.001 (PMC10995970; doi:10.1016/j.asmart.2024.03.001)
Supplement: Multimedia component 1 [file mmc1.pdf]

Editing Certificate

This document certifies that the manuscript listed below has been edited to ensure language and grammar accuracy and is error free in these aspects. The logical presentation of ideas and the structure of the paper were also checked during the editing process. The edit was performed by professional editors at Editage, a brand of Cactus Communications. The author's core research ideas were not altered in any way during the editing process. The quality of the edit has been guaranteed, with the assumption that our suggested changes have been accepted and the text has not been further altered without the knowledge of our editors.

MANUSCRIPT TITLE

The efficacy of periarticular cocktail injection compared with that of nerve blocks for pain control after anterior cruciate ligament reconstruction

AUTHORS

Tomoyuki Kanayama

ISSUED ON

August 08, 2023

JOB CODE

FHJBW_10_7

THE EDITAGE Promise QUALITY GUARANTEE

Vikas Narang

Vikas Narang
Chief Operating Officer - Editage

editage helping you get published

Since 2002, Editage has helped over 430,000 authors publish around 1.2 million research papers in scholarly journals across over 1000 disciplines through editorial, translation, transcription, and publication support services. Editage is a brand of Cactus Communications (cactusglobal.com), a science communication and technology company.

CACTUS.

GLOBAL :
+1(833) 979-0061 | request@editage.com

JAPAN :
0120-50-2987 | submissions@editage.com
